# Role of Fucoxanthin towards Cadmium-induced renal impairment with the antioxidant and anti-lipid peroxide activities

**DOI:** 10.1080/21655979.2021.1973875

**Published:** 2021-09-27

**Authors:** Haoyue Yang, Ronge Xing, Song Liu, Huahua Yu, Pengcheng Li

**Affiliations:** a CAS and Shandong Province Key Laboratory of Experimental Marine Biology, Center for Ocean Mega-Science, Institute of Oceanology, Chinese Academy of Sciences, Qingdao, China; b Laboratory for Marine Drugs and Bioproducts, Pilot National Laboratory for Marine Science and Technology (Qingdao), Qingdao, China

**Keywords:** Apoptosis, cadmium chloride, fucoxanthin, oxidative stress, renal function

## Abstract

Kidney damages caused by cadmium are considered to be one of the most dangerous consequences for the human body. This study aimed to investigate the protective effects of fucoxanthin supplementation on mice models subjected to cadmium-induced kidney damage. The mice treated with cadmium chloride (CdCl_2_) were observed to have significantly reduced the cross-section area of glomeruli. Cadmium exposure has also caused the damage of the structural integrity of mitochondria and increased blood urea nitrogen (BUN), kidney injury molecule 1 (KIM1), and neutrophil gelatinase associated lipocalin (NGAL) levels. Peroxidase (POD), superoxide dismutase (SOD), catalase (CAT), and ascorbate peroxidase (APX) levels in cadmium-exposed mice were markedly declined. Caspase3, caspase8, and caspase9 gene expressions in association with apoptosis were dramatically elevated in renal tissues. The CdCl_2_ treated mice were orally administered with 50 mg/kg Shenfukang, 10 mg/kg, 25 mg/kg, and 50 mg/kg fucoxanthin for 14 days. The results revealed that high doses of fucoxanthin administration significantly decreased BUN, KIM1, NGAL levels, increasing POD, SOD, CAT, and ascorbate APX levels. Fucoxanthin administration also promoted recovery of the renal functions, micro-structural organization, and ultra-structural organization in the renal cells. In summary, the ameliorative effects of fucoxanthin supplementation against cadmium-induced kidney damage were mediated via inhibiting oxidative stress and apoptosis, promoting the recovery of structural integrity of mitochondria.

## Introduction

Cadmium is a potentially toxic heavy metal pollutant in the natural environment [1]. It can be concentrated, accumulated, and amplified in life organisms, water, air, and soil. After cadmium exposure, the human body was reported to develop symptoms of cadmium poisoning. Long-term exposure will result in a higher risk of developing cancer [2,3]. Cadmium has a long half-life (up to 10–25 years), a low excretion rate, and easily accumulated in the body [4]. When the accumulation of cadmium in the human body reaches the toxicity threshold of some organs, serious toxicity and damage symptoms in the organs are present [4]. The main harm of cadmium to the human body is kidney damage. Nearly 50% of the cadmium is accumulated in the kidney, especially in the proximal convoluted tubule, leading to kidney damage and chronic kidney disease [5–8]. Previous studies revealed that up to 7% of the global population suffers from chronic kidney disease caused by cadmium [9]. Therefore, finding a compound for prevention and treatment of cadmium toxicity is a very urgent issue.

Fucoxanthin, also named carotenoid, is a kind of lipopolysaccharide pigment, which is widely distributed in phylum alginate, diatoms, rhodophyceae, dinophyceae, chrysophyceae, and haptophyceae [10,11]. A large number of studies demonstrated that fucoxanthin has a variety of physiological activities and functions, including anti-oxidation, anti-inflammation, anti-obesity, anti-tumor, anti-diabetes, and a lack of toxic side effects [12–16]. So, it is widely used in a variety of fields of food, pharmaceuticals, beauty cosmetics, and so on. Cadmium also exerts toxicity by promoting oxidative damage and apoptosis in cells [17]. Therefore, fucoxanthin as an antioxidant may play a role in the prevention and treatment of cadmium poisoning [8].

To explore the ameliorative effects and mechanism of fucoxanthin against cadmium-induced kidney damage, we utilized Kunming mice as a model for investigating the ameliorative mechanism of fucoxanthin on the kidney in case of cadmium poisoning, through a variety of analyses, including oxidative stress, the physiological damage index, the pathological morphology of the kidney, as well as the study of apoptosis-related signal transduction pathways. We hypothesized that fucoxanthin can restore renal cell functions in CdCl_2_ exposed body via anti-oxidative stress and inhibiting apoptosis. We first generated mice model by CdCl2 injection, then administered a serial doses of fucoxanthin and observed renal functions. This study aimed to address molecular mechanisms of fucoxathin in extenuating cadmium poisoning and provided a theoretical basis for prevention and treatment of cadmium mediated renal damage.

## Materials and Methods

### Materials

The expression levels of extracellular signal-regulated kinase 1 (ERK1), mitogen-activated protein kinase1 (MEK1), eukaryotic translation initiation factor 2A (eIF2a), phosphorylated eukaryotic translation initiation factor2a (p-eIf2α), glucose regulatory protein 78 (GRP78), glucose regulatory protein 94 (GRP94), caspase3, caspase8, and caspase9 genes in mice were determined using the transcription-polymerase chain reaction technique (RT-PCR, Shanghai bioengineering Limited by Share Ltd). Renal convalescent tablets (Shenfukang tablets) were purchased from Jilin Aodong Group Liyuan Pharmaceutical Co. LTD (Dunhua city, Jilin province, China). The main components of Shenfukang tablets (used for the treatment of acute and chronic nephritis) [18] are poria cocos, motherwort, Sophora sophora, white thatch, and agastache. Analytical grade CdCl_2_, fucoxanthin, and other chemicals were purchased from Solarbio Science & Technology Co., Ltd (Beijing, China).

### Experimental Animals and Model Choice

Kunming mice with strong disease resistance, adaptability, and high survival rate were selected as the animal model. Six- to eight-week-old male Kunming mice (weight, 22–26 gram (g)/per mouse) were obtained from the institute of drug inspection of Qingdao. Animals were fed a standard protein diet (22% protein, Mazuri 5E10) and pure water. The animals were kept in an animal facility at standard temperature (22–25°C) and humidity (50%) with a 12 hours light/12 hours dark cycle, and were acclimated to laboratory conditions for 1 week before the conduction of the experiments. All experimental procedures were performed in strict accordance with the recommendations in the Guide for the Care and Use of Laboratory Animals of the Institutional Animal Ethical Committee and the protocols were approved by the Committee on the Ethics of Animal Experiments of the Institute of Oceanology, Chinese Academy of Sciences, Shandong, and China. All efforts were taken to minimize the suffering of the animals. The animals were anesthetized using ether before blood sampling. Laboratory animal quality certificate code is scxk20140001.

### Experimental Design

Total animals (N = 120) were randomly divided into two groups of equal average body weight. The mice of the control group (N = 20) were given pure water only, whereas the animals of the cadmium exposure group (N = 100) were given CdCl_2_ orally at a dose of 30 milligram (mg)/kilogram (kg) body weight (bw)/day for 30 days. In this study, fucoxanthin was administered at 10, 25 and 50 mg/kg bw/day. To evaluate ameliorative effects of fucoxanthin on the kidney, the cadmium exposure group was divided into the following five subgroups: without fucoxanthin treatment as a negative control group (NCG, N = 20); positive control group (PCG, N = 20) was mice received Shenfukang tablets orally at a dose of 50 mg/kg bw/day for 14 days; low (F1), medium (F2), and high (F3) fucoxanthin concentration treated mice (N = 20 mice at each group) were received fucoxanthin orally at a dose of 10, 25, and 50 mg/kg bw/day for 14 days, respectively.

### Animal Sacrifice and Sample Collection

After the 14-day fucoxanthin treatment, mice were sacrificed. Kidney tissues and peripheral blood samples were collected directly. In each group, kidney samples of 14 mice were subsequently kept at −80°C in ultra cold storage freezer for analyses of cadmium concentration, antioxidant activity and relative gene expression; 3 mice were kept at 4°C in 10% neutral-buffered formalin for microscopic observation; and 3 mice were kept at 4°C in 2.5% neutral-buffered glutaraldehyde for electron microscope observation. As for the blood samples, they were added to the test tubes with anticoagulant, centrifuged for 5 minutes at a speed of 1372 g, and the upper plasma was collected for analysis of kidney damage.

### Estimation of Blood Urea Nitrogen (BUN), Kidney Injury Molecule 1 (KIM-1), Neutrophil Gelatinase Associated Lipocalin (NGAL), Peroxidase (POD), Superoxide dismutase (SOD), Catalase (CAT), and ascorbate peroxidase (APX) Levels

BUN levels, POD, SOD, CAT, and APX activities were measured spectrophotometrically with BUN, POD, SOD, CAT, and APX detection kits. KIM-1 and NGAL levels were measured by enzyme-linked immunosorbent assay (ELISA) kits. The BUN kit was purchased from Suzhou Keming Biotechnology Co., LTD (Suzhou city, Jiangsu province, China) and the POD, SOD, CAT and APX kits were purchased from Shanghai Saint-Bio Biotechnology Co., LTD (Shanghai city, China). The ELISA kits were purchased from Shanghai Enzyme-linked Biotechnology Co., LTD (Shanghai city, China). The levels of BUN, KIM-1 and NGAL were expressed as mmol/L, pg/mL and ng/mL. The activities of renal POD, SOD, CAT and APX were expressed as unit (U)/g.

### Total mRNA Extraction and Real-Time Quantitative PCR

In each group, nine frozen kidney samples were used for analysis of relative gene expression. The total RNA was prepared from the kidney tissues using Trizol reagent purchased from Shanghai bioengineering Limited by Share Ltd. Subsequently, mRNA was reverse transcribed to cDNA using oligonucleotide dT primers according to the manufacturer’s instructions (Shanghai bioengineering Limited by Share Ltd). The RT-PCR analyses were subsequently performed using Platinum Taq polymerase and 4S Red Plus nucleic acid stain, both obtained from Shanghai bioengineering Limited by Share Ltd. Primer sequences were designed using the Primer Premier 5.0 software and were listed in revised Table 1. The relative mRNA expression levels were expressed as the value of 2 ^−(∆∆Ct)^ [19].

**Table 1.** Housekeeping Gene (mouse β-actin) and targeted gene expression primer sequences

Gene	Primer sequence	PCR amplicon size
M-β-actin-F	GTGCTATGTTGCTCTAGACTTCG	174bp
M-β -actin-R	ATGCCACAGGATTCCATACC	
M-ERK1-F	GGCTTTCTGACGGAGTATGTG	197bp
M-ERK1-R	GGGGAACCCAAGATACCTAGA	
M- ERK2-F	GGTTGTTCCCAAATGCTGAC	138bp
M- ERK2-R	GCTCATCACTTGGGTCATAATACT	
M- MEK1-F	TGACGCAGAAGCAGAAGGTG	90bp
M-MEK1-R	TGAAGACCACTCCACCGTTG	
M-eIf2a-F	TAATCAATGTCGCTAACAAGGG	152bp
M-eIf2a-R	AAGTTGTAGGTTAGGCGTCCC	
M-p-eIf2a-F	TTCTACAGAAACCATGCCCAT	123bp
M-p-eIf2a-R	TTGATAACTGCCATAGCCTGAT	
M-GRP78-F	GCCAACTGTAACAATCAAGGTCT	234bp
M-GRP78-R	TCAGGTGTCAGGCGGTTTT	
M-GRP94-F	AGGTGTTGTGGATTCCGATGA	225bp
M-GRP94-R	AGTTTAGCAAGCCGTGTTCG	
M-caspase3-F	TGACTGGAAAGCCGAAACTCT	213bp
M-caspase3-R	TGCTGCAAAGGGACTGGAT	
M-caspase8-F	TCAAAGTGCCCTTCCCTGT	197bp
M-caspase8-R	TTCTTCACCGTAGCCATTCC	
M-caspase9-F	AGAACGACCTGACTGCCAAGA	101bp
M-caspase9-R	ATGAGAGAGGATGACCACCACA	

M: mouse; F: forward; R: reverse; ERK1:extracellular signal-regulated kinase 1; MEK1, also named MAP2K1, mitogen-activated protein kinase1; eIF2a, Eukaryotic translation initiation factor 2A;p-IF2a, phosphorylated eukaryotic translation initiation factor2a;GRP78, glucose regulatory protein 78.

### Microstructures and Ultra-microstructures of Renal Tubular Epithelial Cells

After the sacrifice of the mice, kidneys were removed and fixed overnight in 10% neutral-buffered formalin or 2.5% neutral-buffered glutaraldehyde. Fixed tissues were subsequently dehydrated using a graded ethanol series. Tissues that were fixed in 10% neutral-buffered formalin were embedded in paraffin. The sections with 5-μm thickness were cut and mounted on slides, which were then stained with hematoxylin and eosin (H & E), and tissue histopathology was analyzed using a compound microscope (Olympus, BX63). Tissues that were fixed in 2.5% neutral-buffered glutaraldehyde were examined under an electron microscope (Hitachi, H-9500). For the preparation process of electron microscope samples, please refer to the reference [20].

### Semi-quantitative Scoring of Renal Tubular Damage

The histopathological changes of mice kidneys were observed under an optical microscope, and the semi-quantitative scoring of renal tubular damage was carried out according to Erdogan’s method [21]. Each slice was randomly selected under a high-power microscope (400×) for 15 fields, and a 0–4 scoring was conducted for each field. The total score of the 15 fields was subsequently divided by 15, giving the final score of the slice. The Erdogan’s renal tubular damage semi-quantitative scoring standard was as follows. 0 point: no injury; 1 point: minor injury (0–5%); 2 points: mild injury (5–25%); 3 points: moderate injury (25–75%); 4 points: severe injury (75–100%).

### Semi-quantitative Scoring of Mitochondria Damage in Renal Tubular Epithelial Cells

Electron microscopy images were used to conduct a semi-quantitative analysis of renal tissue mitochondria according to the Flameng grading method [22]. Five visual fields were selected from each group. The mitochondria were counted and the degree of damage was scored (0–4 points) in each visual field. Finally, the score of all mitochondria in the five fields was divided by the number of mitochondria, resulting in the final mitochondrial score of the sample. The semi-quantitative scoring standard of the Flameng grading method was as follows. 0 point: mitochondrial morphology and structure were completely normal; 1 point: the mitochondrial structure was normal, part of the mitochondria was slightly swollen and the mitochondrial ridge was separated; 2 points: The mitochondria were severely swollen and the ridge of mitochondria was separated but not broken; 3 points: mitochondria were severely swollen and a large number of mitochondrial ridges were broken; 4 points: mitochondria were vacuolated, extremely swollen, the ridge was broken or even disappeared and the inner and outer membrane was broken.

### Detection of Cadmium Accumulation

In each group, six frozen kidney samples were used for cadmium concentration detection. Renal cadmium accumulation was measured by an inductively coupled plasma emission spectrometer (ICP-OES) and inductively coupled plasma mass spectrometer (ICP-MS). The concentration of cadmium was expressed as ng/mL.

All units of measurement in this section were used following the international system of units (SI) guidelines.

### Statistical Analysis

Data are expressed as means ± standard deviations. Single-factor analysis of variance between multiple groups was performed with Duncan tests. Differences between the means of ≥3 independent experiments were considered significant when P < 0.05. Subsequently, statistical analyses were performed using SPSS 19.0 software.

## Results

### Renal Cadmium Levels Changes in the Kidney

To assess the impacts of cadmium exposure in renal functions, we first detected the basic cadmium level in the kidney of the control group without CdCl_2_ administration. The results were observed to be only 5.28 ± 0.45 ng/mL in the control group (revised Table 2). The CdCl_2_ treatment, but no fucoxanthin as a negative control group (NCG), significantly increased renal cadmium levels (P < 0.05). Treatment with Shenfukang tablets as a positive control greatly decreased the renal cadmium level compared with those in the NCG and 10 (F1) mg/kg bw/day fucoxanthin treatment group (P < 0.05). In contrast, treatments with 25 (F2) and 50 (F3) mg/kg bw/day fucoxanthin blocked the Cd-induced increase in the cadmium level (P < 0.05, compared with the NCG), whereas the renal cadmium levels in the F2 and F3 groups were significantly lower than that of the PCG (P < 0.05). These results indicated that the supplementation with fucoxanthin at 25 or 50 mg/kg bw/day promoted the renal cadmium metabolism, with better amelioration against Cd accumulation than that of the Shenfukang tablets.

**Table 2.** Renal cadmium concentration

Group	Control	NCG	PCG	F1	F2	F3
Cadmium concentration (ng/ml)	5.28 ±0.45	128.55 ±6.75*	92.81 ±3.24*#	120.98 ±9.55*^	79.49 ±6.31*#^	75.49 ±4.56*#^

Single factor analysis of variance between multiple groups was performed using Duncan methods. ‘*’ compared with the control group, P < 0.05; ‘#’ compared with the negative control group, P < 0.05; ‘^’ compared with the positive control group, P < 0.05. Control group: no CdCl_2_ administration; NCG, negative control group, only CdCl_2_ orally at a dose of 30 milligram (mg)/ kilogram (kg) body weight (bw)/day for 30 days; PCG, positive control group, CdCl_2_+ Shenfukang tablets orally at a dose of 50 mg/kg bw/day for 14 days; F1, CdCl_2_+ low fucoxanthin concentration group, mice received fucoxanthin orally at a dose of 10 mg/kg bw/day for 14 days; F2, CdCl_2_+ mice received fucoxanthin orally at a dose of 25 mg/kg bw/day for 14 days; F3, CdCl_2_+ mice received fucoxanthin orally at a dose of 50 mg/kg bw/day for 14 days.

### Microstructures of Renal Cells

**Figure 1.**
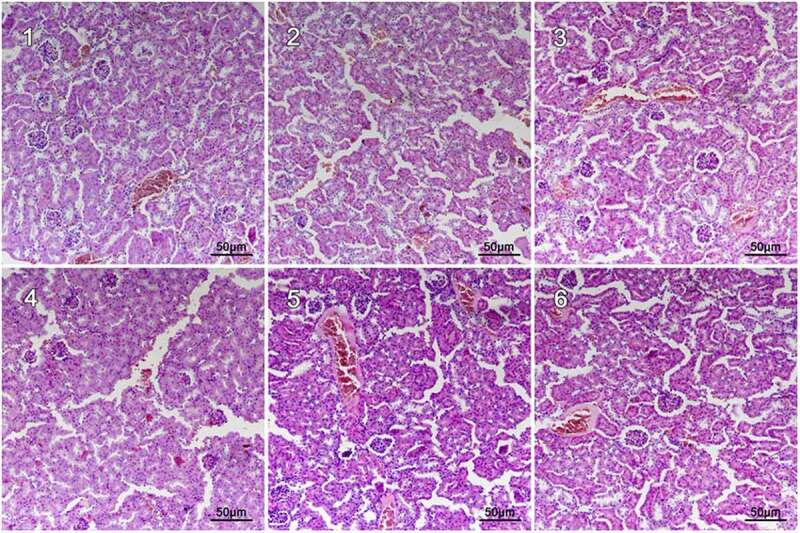
**Hematoxylin and eosin (H &E) staining of renal in CdCl_2_ exposure after the treatment with fucoxanthin and Shenfukang tablets supplementation** (1) the control group: no CdCl_2_ administration; (2) NCG, negative control group, only CdCl_2_ treatment; (3) PCG, positive control group, CdCl_2_+ Shenfukang tablets; (4) F1, CdCl_2_ + 10 mg/kg fucoxanthin body weight for 14 day treatment ; (5) F2, CdCl_2_ + 25 mg/kg body weight fucoxanthin; (6) F3,CdCl_2_ + 50 mg/kg body weight fucoxanthin. Each group used 20 mice

**Figure 2.**
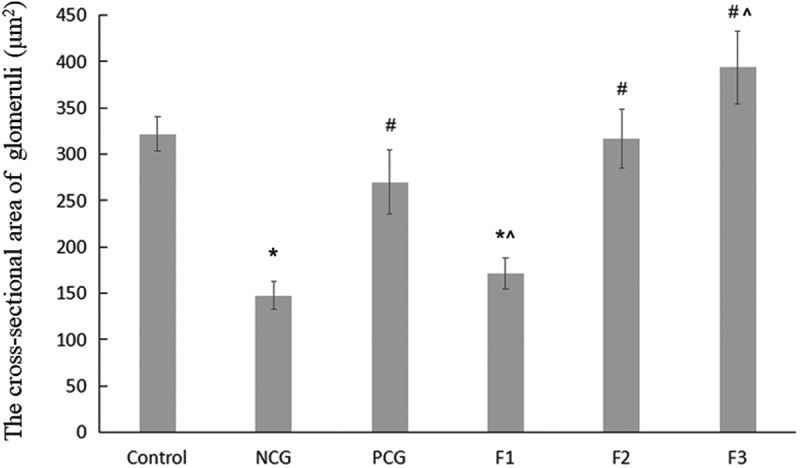
**The quantity of the cross-sectional area of the glomeruli (μm^2^)** Single factor analysis of variance between multiple groups was performed using Duncan methods. ‘*’ compared with the control group, P < 0.05; ‘#’ compared with the negative control group, P < 0.05; ‘^’ compared with the positive control group, P < 0.05. Each group used 20 mice

**Figure 3.**
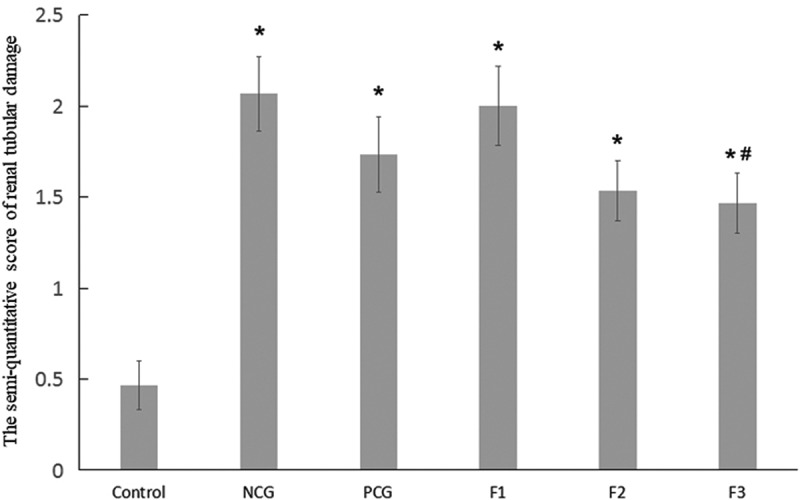
**The semi-quantitative score of renal tubular damage in mice** Single factor analysis of variance between multiple groups was performed using Duncan methods. ‘*’ compared with the control group, P < 0.05; ‘#’ compared with the negative control group, P < 0.05. Each group used 20 mice

To further investigate the relationship between structures and functions of renal in cadmium-exposed mice, we performed hematoxylin and eosin (H & E) staining of renal in CdCl_2_ exposed mice. In the control group, no obvious renal lesions were found. The renal tubules were evenly distributed and closely arranged, as well as the glomeruli were regular round or oval in structure in the control group without CdCl_2_ treatment (Figure 1). The CdCl_2_ exposure in the NCG resulted in obvious pathological damage to the renal tubular epithelial cells and the glomerular structure (Figure 1). The renal tubular epithelial cells were swollen and damaged. The lumen of some renal tubules was dilated, loosely arranged, and presented with necrotic exfoliated cells and tubules. The glomerulus was atrophic and deformed, and its cross-sectional area was significantly lower than that of the control group (P < 0.05) (Figure 1). The pathological damage caused by cadmium exposure was reduced after treatment with Shenfukang tablets and medium as well as high dosage of fucoxanthin (Figure 1). We further compared the changes of kidney microstructure in Shenfukang and different doses of fucoxanthin (Figure 1, Figure 1, and Figure 1). In the PCG (Figure 1), F2 (Figure 1), and F3 (Figure 1) groups, the lesions of the renal tubular epithelial cells were significantly improved, and the morphology of the glomerulus tended to be normal. In contrast, low-dose fucoxanthin treatment showed that the cross-sectional area was not significantly different from that of the control group (Figure 1) . We also performed the quantity of the cross-sectional area of the glomeruli (μm^2^) and semi-quantitative score of renal tubular damage in mice following Erdogan’s methods [21]. The results showed that the cross-sectional area of the glomerulus in NCG was significantly lower than that of the control group (P < 0.05). The pathological damage caused by cadmium exposure was dramatically reduced after treatment with shenfukang tablets and high dosages of fucoxanthin. In the PCG, F2 and F3 groups, the lesions of the renal tubular epithelial cells were significantly improved, the cross-sectional area was not significantly different from that of the control group (P > 0.05) (revised Figure 2). We also carried out the semi-quantitative score of the renal tubular injury in mice and found that the semi-quantitative score of mice in CdCl_2_ exposure was significantly higher than that in the control group (P < 0.05). The semi-quantitative score in the F3 group was significantly lower than that in NCG (P < 0.05) (revised Figure 3). All together, the semi-quantitative score combined with histological observation showed that fucoxanthin had a certain therapeutic effect on renal lesions caused by cadmium exposure.

### Ultra-structures of Renal Tubular Epithelial Cells

**Figure 4.**
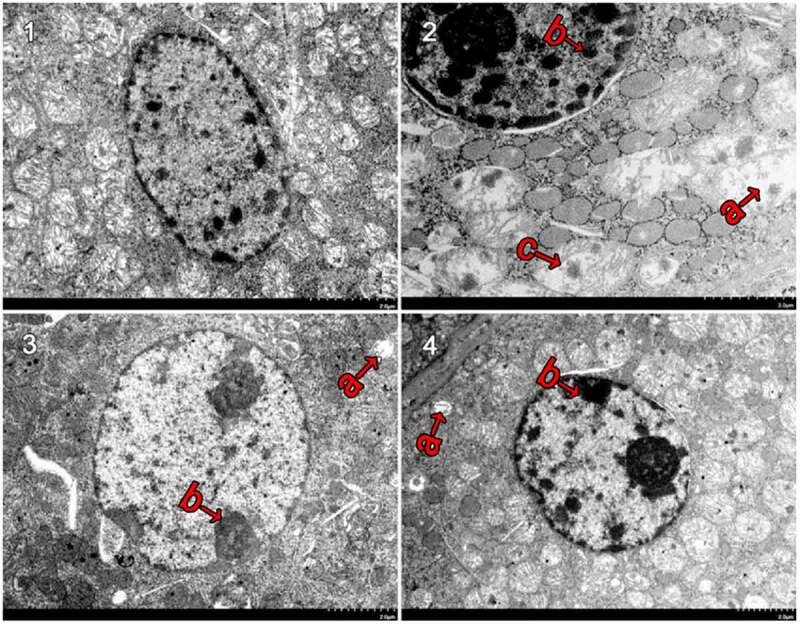
**Ultrastructural changes after treatment with fucoxanthin and Shenfukang tablets administration** (1) Control group, (2) NCG, (3) PCG, (4) F3, (a) mitochondria damage, (b) karyopyknosis, and (c) mitochondrial rupture. Each group used 20 mice

**Figure 5.**
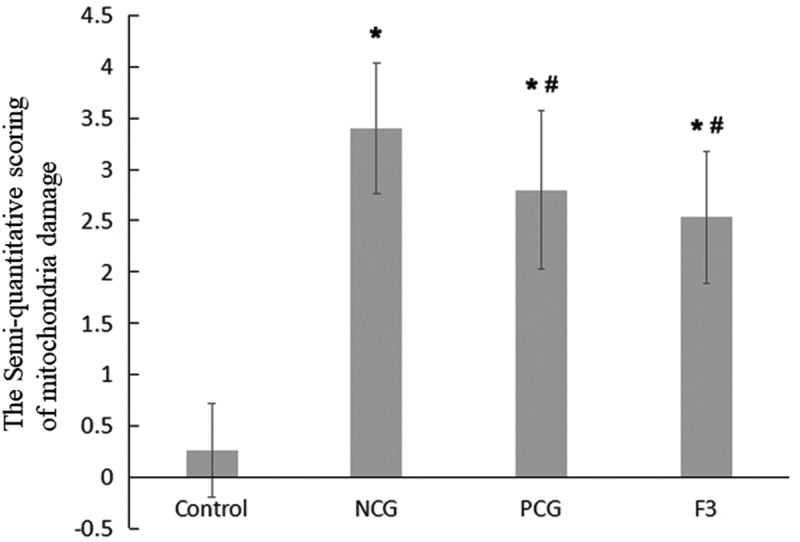
**Semi-quantitative evaluation of mitochondrial damage in mouse kidney cells** Single factor analysis of variance between multiple groups was performed using Duncan methods.‘*’ compared with the control group, P < 0.05; ‘#’ compared with the negative control group, P < 0.05. Each group used 20 mice

Mitochondria are very important organelle in cellular physiology. We speculated that the structure of mitochondria may be damaged. Under an electron microscope, the mitochondria of CaCl_2_-treated renal tubular epithelial cells were observed to be intact, the inner cristae were found to be closely arranged and visible, the nucleus was regular ovoid and there was no obvious karyopyknosis (revised Figure 4). In comparison with the control group, the NCG (revised Figure 4) had more mitochondrial vacuoles degenerate (revised Figure 4) and more mitochondrial cristae disappear (revised Figure 4) or ruptured. In severe cases, the mitochondrial inner and outer membrane ruptured (revised Figure 4). The NCG had a significant increase in the semi-quantitative score of mitochondrial damage (P < 0.05, revised Figure 5). Moreover, the nucleus was slightly deformed and showed obvious karyopyknosis. In comparison with the NCG, the organelle damages were significantly ameliorated in the PCG (revised Figure 4) and the F3 group (revised Figure 4), with only a small amount of mitochondrial vacuolar degeneration, reduced mitochondrial cistae fracture, rare matrix coagulation, and a significantly reduced semi-quantitative score of the mitochondrial damage (P < 0.05, revised Figure 5). In addition, karyopyknosis was found to be significantly reduced.

### Changes in Levels of Kidney Injury Markers

To explore the molecular mechanisms of these physiological changes, we investigated kidney injury relevant biomarkers (revised Table 3). The result showed that the plasma BUN, KIM-1, and NGAL levels of in the CdCl_2_ treated group (NCG) were significantly increased than that in no CdCl_2_ treated control group (P < 0.05). Shenfukang tablets (PCG), 25 (F2) and 50 (F3) mg/kg bw/day of fucoxanthin significantly decreased the BUN levels in comparison with that in the NCG (P < 0.05). BUN levels in F2 group, F3 group and PCG group were not significantly different from those in the control group (P > 0.05). Shenfukang tablets and fucoxanthin treatments significantly decreased the KIM-1 level in comparison to the NCG (P < 0.05). The KIM-1 level in the F2 group was not significantly different from that in the control group (P > 0.05), indicating that the 25 mg/kg bw/day of fucoxanthin supplementation could decrease the KIM-1 level toward normal value. Shenfukang tablets and fucoxanthin treatments significantly decreased the NGAL levels in comparison with those in the NCG (P < 0.05). The NGAL level in the PCG group was not significantly different from that in the control group (P > 0.05), indicating that the Shenfukang tablets supplementation could decrease the NGAL level toward normal value.

**Table 3.** Changes in the BUN, KIM-1 and NGAL levels

	Control	NCG	PCG	F1	F2	F3
BUN (mmol/L)	2.70 ±0.12	4.13 ±0.16*	2.94 ±0.11#	4.35 ±0.20*^	2.64 ±0.16#	2.55 ±0.20#
KIM-1 (pg/ml)	67.67 ±35.69	713.86 ±117.82*	473.67 ±97.94*#	404.92 ±73.13*#	174.85 ±87.75#^	281.07 ±79.56*#^
NGAL (ng/ml)	39.61 ±9.66	95.52 ±9.38*	45.77 ±9.80#	76.70 ±13.19*#^	63.60 ±6.08*#^	60.85 ±12.76*#^

Single factor analysis of variance between multiple groups was performed using Duncan methods. ‘*’ compared with the control group, P < 0.05; ‘#’ compared with the negative control group, P < 0.05; ‘^’ compared with the positive control group, P < 0.05. Control group: no CdCl_2_ administration; NCG, negative control group, only CdCl_2_ orally at a dose of 30 milligram (mg)/ kilogram (kg) body weight (bw) /day for 30 days; PCG, positive control group, CdCl_2_+ Shenfukang tablets orally at a dose of 50 mg/kg bw/day for 14 days; F1, CdCl_2_+ fucoxanthin orally at a dose of 10 mg/kg bw/day for 14 days; F2, CdCl_2_+ fucoxanthin orally at a dose of 25 mg/kg bw/day for 14 days; F3, CdCl_2_+ fucoxanthin orally at a dose of 50 mg/kg bw/day for 14 days.

### Effects of Fucoxanthin on Antioxidant in CdCl_2_-treated Mice

**Figure 6.**
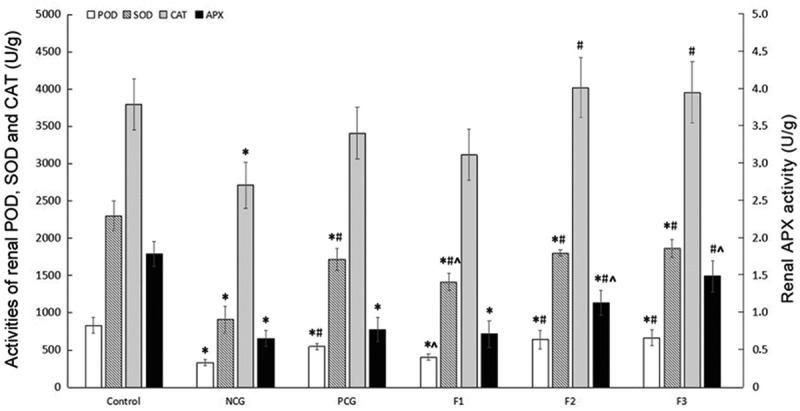
**Changes in Renal POD, SOD, CAT and APX Activities (U/g)** Single factor analysis of variance between multiple groups was performed using Duncan methods. ‘*’ compared with the control group, P < 0.05; ‘#’ compared with the negative control group, P < 0.05; ‘^’ compared with the positive control group, P < 0.05. Each group used 20 mice

To assess the roles of fucoxanthin in anti-oxidative stress, we detected a few key enzyme levels in lipid peroxidation pathways. Activities of renal POD, SOD, CAT, and APX in the NCG mice were significantly decreased, compared with those in the control group (P < 0.05). Fucoxanthin and Shenfukang tablets treatments significantly improved redox states compared with that in mice of the NCG. Mice treated with middle (F2) and high (F3) concentrations of fucoxanthin administration showed significant improvement in POD, SOD, CAT and APX activities compared to the control group. There were no significant differences in renal CAT and APX activities between the PCG and NCG (P > 0.05). In the F2 and F3 groups, the renal CAT activity was significantly higher than that of NCG (P < 0.05), and there was no significant difference compared with the control group (P > 0.05). The renal APX activity in the F2 and F3 groups was significantly higher than that of NCG (P < 0.05), but in F3, there was no significant difference compared with the control group (P > 0.05), indicating that 25 and 50 mg/kg bw/day of fucoxanthin treatment exerted a better effect in increasing CAT and APX activities than Shenfukang tablets (Figure 6).

### Molecular Mechanisms Underlying the Therapeutic Effects of Fucoxanthin against Cell Death

**Figure 7.**
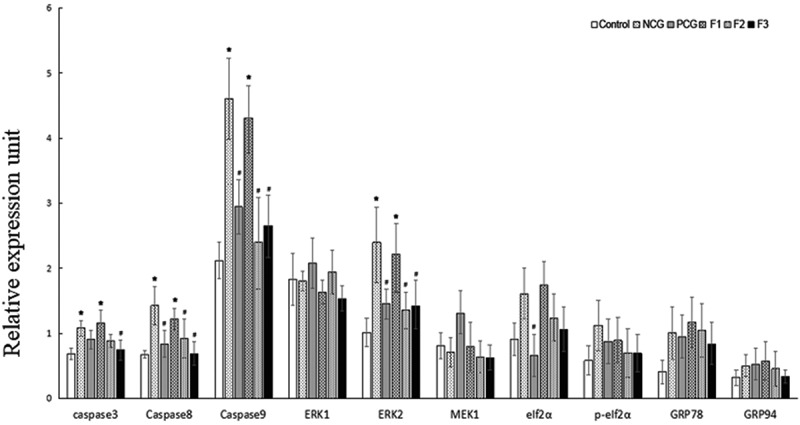
**Effect of fucoxanthin on the expression of apoptosis-associated genes in the cadmium kidney-damaged mouse model** Single factor analysis of variance between multiple groups was performed using Duncan methods.‘*’ compared with the control group, P < 0.05; ‘#’ compared with the negative control group, P < 0.05. Each group used 20 mice

To address roles of fucoxanthin in cell apoptosis, we measured the expression levels of ERK1, ERK2, MEK1, eIf2α, p-eIf2α, GRP78, GRP94, caspase3, caspase8 and caspase9 mRNA in mice (revised Figure 7). Compared with the control group, the relative mRNA level of the caspase 3 was strongly up-regulated in the NCG (P < 0.05), and this transcript was strongly down-regulated in the 50 mg/kg bw/day (F3) fucoxanthin treatment group compared with that in the NCG (P < 0.05). Compared with the control group, the relative mRNA levels of the caspase 8, the caspase 9, and the ERK2 were strongly up-regulated in the NCG (P < 0.05), and these transcripts were strongly down-regulated in the PCG, 25 (F2), and 50 mg/kg bw/day (F3) fucoxanthin treatment groups compared with those in the NCG (P < 0.05). Compared with the control group, the relative mRNA expression levels of eIf2α, p-eIf2α, GRP78 and GRP94 were all up-regulated in the NCG, but did not differ significantly between the NCG and fucoxanthin groups (P > 0.05).

## Discussion

In recent years, fucoxanthin has been widely investigated due to its powerful antioxidant and free radical scavenging ability [23–25]. The apoptosis induced by oxidative stress is one of the primary mechanisms that are in place in regard to the toxic effects of the heavy metal cadmium [26,27]. On the other hand, few reports were published previously on the ameliorative effect of fucoxanthin against the cadmium-induced toxic effects [8,28,29]. Therefore, this study investigated the ameliorative effects of fucoxanthin on cadmium-exposed kidneys in mice to provide theoretical reference for preventing and relieving heavy metal toxicity as well as the further development of fucoxanthin.

The studies indicated that after entering the organism, cadmium binds to metallothionein to form a cadmium-metallothionein complex (CD-MT) [30,31]. The CD-MT is transported to the kidney through blood, subsequently filtered through glomerular filtration, and enters the renal tubular through pinocytosis and is isolated by lysosomes in renal tubular epithelial cells to release free cadmium, which is eventually deposited in the kidney [5]. Liu et al. [32] study revealed that after chronic cadmium poisoning in mice, most of the cadmium was deposited in proximal convoluted tubules, leading to their degeneration. Bharathiraja et al. [8] reported that BUN, creatinine, and lipid peroxidation levels of serum in CdCl_2_ treated rat model were significantly increased and SOD, POD, and reduced glutathione were dramatically declined in CaCl_2_ exposed rat model. They also found that administration of fucoxanthin in CaCl_2_ treated rats significantly reduced the levels of blood urea nitrogen, creatinine, lipid peroxidation and elevated the levels of SOD, POD, and reduced glutathione. Indeed, our results confirmed these findings and further identified that fucoxanthin can play its role via recovering structure of mitochondria and inhibiting apoptosis of renal cells. In addition, 25–50 mg/kg bw/day of fucoxanthin supplementation was superior to the Shenfukang tablets, which can significantly reduce the accumulation of cadmium in the mice kidneys, restore the structural integrity of the renal tubules and glomeruli, and ameliorate the damage of the renal tissue.

Cadmium can enter the viscera cells through simple diffusion, calcium ion channels or other transport systems, and induce the generation of free radicals and reactive oxygen species to trigger oxidative stress, which may be one of the main mechanisms of cadmium-induced organ damage [33]. Therefore, the inhibition of oxidative stress damage may play a key role in the prevention of cadmium toxicity. Oxidative stress can be prevented by antioxidant enzymes and antioxidants. The antioxidant defense system enzymes include SOD, APX, POD, CAT, as well as other enzymes. Our previous study revealed that administration of fucoxanthin in CdCl_2_ exposed thyroid follicle cells restored thyroid functions via inhibiting the ERK1/2 pathway and restraining endoplasmic reticulum stress [28]. Here, our data have shown that the cadmium can significantly decrease POD, SOD, CAT, and APX activities in mice kidney, indicating that cadmium causes oxidative stress. The fucoxanthin treatment can restore these antioxidant indicators listed above to normal levels, ameliorate organism antioxidant capacity and alleviate oxidative stress damage caused by cadmium. Combined with previous studies on the antioxidant properties of fucoxanthin, it is believed that the antioxidant activity of fucoxanthin is mainly reflected in its free radical scavenging ability, which is related to the presence of oxygen atoms in the molecule. The fucoxanthin molecule contains six oxygen atoms and a propylene structure, which makes it more sensitive to free radicals and possess strong antioxidant activity. Furthermore, fucoxanthin prevents lipid peroxidation by decreasing the activity of the Na+K+ -ATPase as well as increasing the activity of catalase and glutathione transferase [34]. This is consistent with the results observed by microscope and electron microscope for that medium- and high-dose fucoxanthin was good at ameliorating the cadmium-induced cell damage. The most prominent contribution of fucoxanthin is to maintain the structural and morphological integrity of mitochondria.

The effect of cadmium on the mitochondria has been considered as the main cause of cell apoptosis [35,36]. Regarding the mechanism of cell apoptosis, abnormal mitochondrial function is an important pathway [37,38]. The damage of mitochondrial inner membrane can be accompanied by the release of apoptotic induction factor and Caspase proteinase in the mitochondrial membrane gap, which causes apoptosis through the cascade reaction of apoptosis [39]. Our results showed that the cadmium ions could significantly promote the expression of Caspase3, Caspase 8 and Caspase 9 in mice kidneys. After Shenfukang tablets, 25 and 50 mg/kg bw/day fucoxanthin treatment, the expressions of Caspase3, Caspase 8 and Caspase 9 decreased and there was no significant difference in comparison with the control group. These results confirmed that fucoxanthin may ameliorate the renal damage caused by cadmium by reducing oxidative stress damage in the kidney, maintaining mitochondrial structure integrity, and regulating the expression of Caspase3, Caspase 8, and Caspase 9 to inhibit cell apoptosis in kidney cells.

Blood BUN is a common index to judge glomerular filtration function [40]. In renal insufficiency, BUN level was increased. KIM-1 is derived from renal tubular epithelial cells. Its expression is enhanced when epithelial cells are injured and continues until epithelial cells get repaired [41]. NGAL is a microprotein expressed by neutrophils and renal tubular epithelial cells. In ischemic or nephrotoxic kidney injury, NGAL is expressed in large quantities in kidneys and is released into urine and plasma. Our results showed that the concentration of cadmium and the levels of BUN, KIM-1 and NGAL in cadmium-exposed mice were significantly increased, confirming that cadmium ion could accumulate in the kidney and lead to kidney damage that can disrupt its function. Supplementation of 25–50 mg/kg bw/day of fucoxanthin can significantly promote the excretion of cadmium and reduce the levels of BUN, KIM-1 and NGAL in cadmium poisoned mice, indicating that fucoxanthin can ameliorate the renal function, promote the recovery of renal tubular epithelial cells and the glomerular filtration rate, and accelerate kidney detoxification.

## Conclusion

Fucoxanthin exerted therapeutic effects via inhibiting oxidative stress and apoptosis in CdCl_2_ exposed kidneys. The effective mechanisms of fucoxanthin are mediated by restoring the structural intact of mitochondria and increasing the antioxidant capacity of the body. These data provided the foundation for the clinical treatment of fucoxanthin in the cadmium-exposed patients with kidney dysfunctions.

## Data Availability

Data in this study is available from the corresponding author on request.

## Highlights

- Cadmium chloride can trigger oxidative stress and promote apoptosis.
- Cadmium chloride can damage structural integrity of mitochondria in renal cells.
- Fucoxanthin decreased the oxidative stress and apoptosis in cadmium chloride-induced mice models.

Fucoxanthin may treat kidney dysfunctions caused by cadmium exposure.
